# Association between Daily Dietary Calcium Intake and the Risk of Cardiovascular Disease (CVD) in Postmenopausal Korean Women

**DOI:** 10.3390/nu16071043

**Published:** 2024-04-03

**Authors:** Jae Kyung Lee, Thi Minh Chau Tran, Euna Choi, Jinkyung Baek, Hae-Rim Kim, Heeyon Kim, Bo Hyon Yun, Seok Kyo Seo

**Affiliations:** 1 Department of Obstetrics and Gynecology, Severance Hospital, Yonsei College of Medicine, 50-1 Yonsei-ro, Seodaemun-gu, Seoul 03722, Republic of Korea; jjackielee@yuhs.ac (J.K.L.); euna_ya@yuhs.ac (E.C.); nupy90@yuhs.ac (J.B.); kimhy@yuhs.ac (H.K.);; 2 Department of Obstetrics and Gynecology, Tu Du Hospital, University of Medicine and Pharmacy, Ho Chi Minh City 700000, Vietnam; drchautran@ump.edu.vn; 3 College of Natural Science, School of Statistics, University of Seoul, 163 Seoulsiripdae-ro, Dongdaemun-gu, Seoul 02504, Republic of Korea; haley2203@naver.com

**Keywords:** menopause, postmenopausal women, cardiovascular diseases, dietary calcium intake, cross-sectional study, Korea national health and nutrition examination survey (KNHANES)

## Abstract

We aimed to evaluate the association between daily dietary calcium intake and the risk of cardiovascular disease (CVD) in postmenopausal women using data from the Korean National Health and Nutrition Examination Survey (KNHANES). This cross-sectional study included 12,348 women aged 45–70 years who had reached natural menopause. They were classified into three groups according to daily dietary calcium intake: <400 mg, 400–800 mg, and >800 mg. The risks of CVD, stroke, angina, and myocardial infarction were assessed in each group. Further, we performed subgroup analysis according to the post-menopause duration (≤10 vs. >10 postmenopausal years). We performed logistic regression analysis with adjustment for age, menopausal age, income, urban area, education, insulin use, body mass index, hypertension, diabetes mellitus, dyslipidemia, high alcohol intake, smoking, exercise, oral contraceptive use, and hormonal therapy use. Calcium intake level was not significantly associated with the risk of CVD in the total population and the ≤10 postmenopausal years subgroup. However, in the >10 postmenopausal years subgroup, daily calcium intake >800 mg was associated with significantly decreased risks of all CVD (odds ratio [OR], 0.27; 95% confidence interval [CI], 0.11–0.64), stroke (OR, 0.06; 95% CI, 0.01–0.42), and myocardial infarction (OR, 0.27; 95% CI, 0.11–0.64). Our findings suggest that a dietary calcium intake of >800 mg/day decreases the risk of CVD events in women who have been menopausal for >10 years.

## 1. Introduction

Calcium is an essential element of human physiology. It enhances bone density, muscle contraction, signal transmission, and vascular tone [1]. Adequate calcium intake is crucially involved in ensuring bone health, especially in postmenopausal women [2,3]. Low dietary calcium intake is associated with many adverse effects, including osteoporosis and fracture risk. However, high calcium intake has been associated with an increased risk of cardiovascular disease (CVD) [4]. Since the 2008 Auckland Calcium Study, there have been concerns about the adverse effects of high-calcium supplements with respect to CVD [5], with subsequent studies demonstrating that high calcium intake might increase the risk of CVD [6,7,8,9,10]. The proposed mechanism for calcium affecting CVD is that increased circulating calcium could increase vascular smooth muscle contraction, blood pressure, and hypertension. It might also increase coagulability or might change vascular flow and thrombotic events. Another potential adverse effect is increasing coronary artery calcification as well as coronary and carotid artery plaques [1,6].

Contrastingly, several studies showed that calcium intake was not associated with CVD [11,12]. Other studies have shown that neither dietary nor supplemental calcium intake was associated with death caused by CVD, heart disease, or cerebrovascular disease among women over fifty years old [13,14]. Therefore, it remains unclear whether calcium intake is associated with the risk of CVD. Numerous clinical studies have reported a beneficial effect of calcium intake on CVD, with increased dietary calcium intake being associated with a decreased risk of CVD [15,16,17,18]. A meta-analysis in 2020 emphasized that dietary calcium intake did not increase the risk of CVD, while supplemental calcium intake increased the risk of myocardial infarction (MI) and CVD [19]. Another meta-analysis in 2023 showed that calcium intake alone or with vitamin D was not associated with CVD [20].

Based on the American Association of Clinical Endocrinologists (AACE) and the United States Institute of Medicine (IOM), the recommended dietary allowance is 1000 mg/day for premenopausal women and 1200 mg/day for women over fifty years old [21,22]. However, calcium consumption in Korean women has been shown to be much lower than recommended. A large prospective Korean cohort study reported that the mean daily calcium intake was ≈500 mg/day [14]. Therefore, it is important to evaluate cardiovascular risk according to calcium intake in the Korean female population. Accordingly, this study aimed to evaluate the association between daily calcium intake and CVD risk in postmenopausal Korean women using data from the Korean National Health and Nutrition Examination Survey (KNHANES).

## 2. Materials and Methods

### 2.1. Participants

The KNHANES was conducted by the Korea Disease Control and Prevention Agency, recruiting ≈10,000 representative non-institutionalized civilians aged ≥1 year since 1998. In this study, we analyzed survey data collected from 2005 to 2021. This survey was conducted at 3-year intervals until 2007 and annually thereafter. The survey included a health interview, nutrition examination, and health examination (physical measurements, laboratory test results, bone mineral density, and body mass index [BMI]). Trained interviewers conducted health interviews and health examinations during home visits, and the surveys were completed by the participants or through an interview format. Subsequently, a trained dietitian conducted nutritional surveys using a 24 h dietary recall and a food frequency questionnaire.

**Figure 1:**
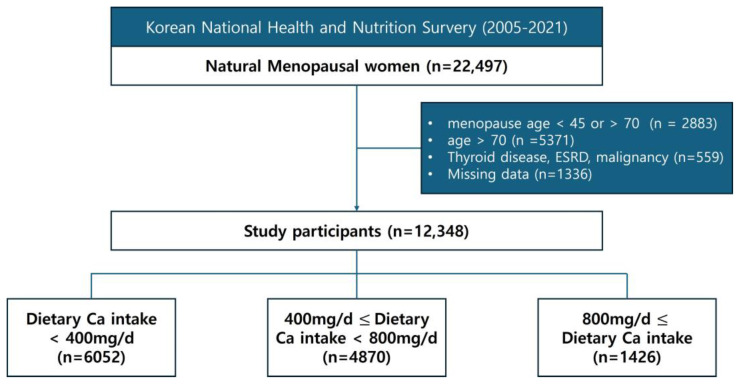
Flowchart of participants.

Figure 1 shows a flowchart of the participants. We excluded women with menopause due to iatrogenic causes, including bilateral salpingo-oophorectomy, hysterectomy, radiation therapy, or chemotherapy. Among the 22,497 women who had natural menopause, we excluded 2883 women who had menopause before and after the age of 45 and 70 years, respectively. Additionally, we excluded 5371 women aged >70 years and 1336 women with missing data regarding calcium intake. Finally, we excluded 559 women with thyroid dysfunction, end-stage renal disease, and malignancy, which may involve altered calcium metabolism. Ultimately, 12,348 participants were included in this study.

### 2.2. Data Collection

Clinical data were collected from standardized questionnaires, including the participants’ menarche age, menopausal age, smoking history, exercise frequency, alcohol consumption, history of hormone replacement therapy, history of oral contraceptive use, history of hypertension, diabetes mellitus, education, income, and daily calcium intake.

Menopause was determined retrospectively after 12 consecutive months of amenorrhea after the final menstrual period [23]. Based on the criteria presented by the Korea Centers for Disease Control and Prevention, terms for hypertensive disorders and diabetic disorders were defined as follows. Hypertension was defined as systolic blood pressure (SBP)  ≥  140 mmHg, diastolic blood pressure (DBP)  ≥  90 mmHg, or self-reported use of antihypertensive medication. Pre-hypertensive stage was defined as SBP between 120 mmHg and 139 mmHg and DBP between 80 mmHg and 89 mmHg. Normotension was defined as SBP less than 120 mmHg and DBP less than 80 mmHg. Diabetes patients were defined as participants with previous diagnosis by a doctor based on a self-report or based on the survey results, with fasting plasma glucose (FPG) levels ≥126 mg/dL (7.0 mmol/L) and/or HbA1c levels ≥6.5%. FPG disorder was defined as FPG levels of 100–125 mg/dL (5.6–6.9 mmol/L).

Further, self-reported medical history, including history of stroke, angina, and MI, was obtained. Exercise was defined as physical activity characterized by moderate or vigorous exercise for >20 min. High alcohol intake was defined by the Korean Ministry of Health and Welfare as drinking more than seven servings (male) or five servings (female) of alcohol more than twice a week.

Daily calcium intake was determined as the sum of the amounts of calcium contained in the foods consumed individually per day. Daily energy and nutrient intake were calculated using the Korean Food Composition Database of the Rural Development Administration. This study was approved by the Institutional Review Board of Severance Hospital, Yonsei University College of Medicine (approval No. 4-2023-1382, 13 December 2023).

### 2.3. Statistical Analysis

Continuous and categorical variables are presented as weighted mean (±standard error) and weighted row percent (standard error), respectively. Categorical variables were analyzed using the chi-squared test. Analysis of variance was used to compare baseline characteristics according to dietary calcium levels. Logistic regression analysis was performed to evaluate the odds ratios (ORs) among the different calcium intake groups. The SURVEYFREQ, SURVEYMEANS, and SURVEYREG SURVEYLOGISTIC were used to calculate the statistics. All statistical analyses and visualizations were performed using R (version 4.2.1; The R Foundation, www.R-project.org) and SAS version 9.4 (SAS Institute Inc., Cary, NC, USA). Statistical significance was set at a two-sided *p*-value < 0.05.

## 3. Results

Table 1 presents the clinical characteristics of the study participants. Among the 12,348 included participants, 6052, 4870, and 1426 women had daily calcium intake <400 mg/day, 400–800 mg/day, and >800 mg/day, respectively. Only 338 (2.7%) women consumed more calcium per day than the intake recommended by the North American Menopause Society, which is 1200 mg/day for women over fifty years old. Three groups were established based on the daily dietary calcium intake: <400 mg, 400–800 mg, and >800 mg. The risks of CVD, stroke, angina, and MI were assessed in each group. Subgroup analysis was performed according to the postmenopausal duration (≤10 vs. >10 postmenopausal years).

The average age of the women showed a trend of decreasing with increasing dietary calcium intake, as follows: 59.58 (±0.1), 58.76 (±0.1), and 58.71 (±0.18) years for daily calcium intake <400 mg/day, 400~800 mg/day, and >800 mg/day, respectively. The ages at menopause showed no significant among-group differences (50.73 [±0.05], 50.79 [±0.05], and 50.78 [±0.09] years for the corresponding groups [*p* = 0.43]).

Regarding socioeconomic factors, daily calcium intake was positively correlated with education level, income level, and urban residence. Moreover, daily calcium intake was negatively correlated with BMI and the prevalence of comorbidities, including hypertension, diabetes mellitus, and dyslipidemia. Moreover, dyslipidemia and insulin use were less prevalent in populations with high calcium intake. However, oral contraceptive use did not significantly differ among the groups. Women with a calcium intake of 400–800 mg/day had the highest percentage of hormonal therapy compared with the other calcium intake groups. Regarding lifestyle parameters, higher daily calcium intake was associated with less excessive alcohol consumption and more frequent physical exercise; however, the daily calcium intake was not associated with smoking history.

Logistic regression analysis was performed to minimize the effects of differences in baseline characteristics. Model 1 was adjusted for age, while Model 2 was adjusted for age, menopausal age, income, urban area, education, insulin usage, BMI, hypertension, diabetes mellitus, dyslipidemia, high alcohol intake, smoking, exercise, oral contraceptive use, and hormonal therapy usage.

Table 2 shows the association of daily calcium intake with the risk of CVD in all women (age: 45–70 years). CVDs were classified as stroke, angina, and MI. In the unadjusted analysis, in women with a daily calcium intake of 400–800 mg, there were significantly decreased ORs for all CVD (OR, 0.75; 95% CI, 0.61–0.92) and angina (OR, 0.57; 95% CI, 0.33–0.99). Additionally, in women with daily calcium intake > 800 mg, there were significantly decreased ORs for stroke (OR, 0.57; 95% CI, 0.35–0.92) and angina (OR, 0.31; 95% CI, 0.1–0.91). However, the adjusted OR showed that calcium intake at any level did not affect the risk of CVD events in women aged 45–70 years.

Table 3 depicts the risk of CVD in each daily calcium intake group for women with <10 years of postmenopausal status. In Model 2, women with daily calcium intake >400 and ≤800 mg showed non-significantly decreased ORs for all CVDs (OR, 0.81; 95% CI, 0.49–1.36). In women with daily calcium intake >800 mg, there was a non-significantly decreased OR for all CVDs (OR, 1.81; 95% CI, 0.87–3.76). Calcium intake at any level did not affect the risk of CVD events in women who had been postmenopausal for <10 years.

Table 4 shows the risk of CVD in each daily calcium intake group for women with >10 years of postmenopausal status. In women with a daily calcium intake of >800 mg, the unadjusted model showed a reduced risk for all CVDs (OR, 0.46; 95% CI, 0.26–0.8), stroke (OR, 0.39; 95% CI, 0.2–0.78), and MI (OR, 0.47; 95% CI, 0.23–0.98). Adjusted multivariable analysis revealed significantly decreased risks of all CVDs (OR, 0.27; 95% CI, 0.11–0.64), stroke (OR, 0.06; 95% CI, 0.01-0.42), and MI (OR, 0.27; 95% CI, 0.11-0.64) with daily calcium intake >800 mg.

## 4. Discussion

This study demonstrated that women with menopausal duration >10 years, but not ≤10 years, experienced a reduced risk of CVD events, stroke, and MI when their daily calcium intake exceeded 800 mg. Our findings are consistent with previous reports that increased dietary calcium intake is associated with decreased CVD risk. A prospective cohort study of 74,245 women aged 30–55 years in the Nurses’ Health Study reported a negative correlation between calcium consumption and CVD risk. After 24 years of follow-up, there was a decreased risk of coronary artery disease in women with a daily calcium intake >1000 mg [24]. A Korean study reported that in women over fifty years old, increased dietary calcium consumption was associated with a decreased CVD risk [16], especially in women without obesity [15]. Other studies have reported that neither dietary nor supplemental calcium intake was associated with death caused by CVD, heart disease, or cerebrovascular disease in women over fifty years old [13,14]. A meta-analysis in 2020 emphasized that dietary calcium intake did not increase the risk of CVD but that calcium supplementation might increase the risk of MI and CVD [20].

Several mechanisms may explain the beneficial effect of appropriate calcium intake on the risk of CVD among women who have been menopausal for >10 years. Calcium intake may have beneficial effects in circulating lipids in postmenopausal women by increasing HDL and decreasing LDL [25]. A small increase in serum calcium may lead to a transient decrease in PTH and may not have a harmful cardiovascular effect [26]. Hypercalcemia and a decrease in PTH may be associated with decreased blood pressure [27,28]. Calcium intake may increase insulin sensitivity [29]. Estrogen is crucially involved in regulating intracellular calcium homeostasis [8]. By regulating the release of calcium from the sarcoplasmic reticulum and limiting calcium overload, it prevents cardiovascular diseases caused by elevated calcium levels in women. Additionally, it decreases the prevalence of cardiovascular diseases associated with cardiac diastolic dysfunction in postmenopausal women. Long-term estrogen withdrawal alters calcium homeostasis-related proteins (L-type Ca^2+^ channel, ryanodine receptor, sarco(endo)plasmic reticulum Ca^2+^-ATPase, and sodium–calcium exchanger) in cardiomyocytes [30]. Changes in the calcium homeostasis cycle are major risk factors for cardiac systolic and diastolic dysfunction as well as the mechanism of CVD. The effect of calcium on CVD seems to vary across different menopause stages. Moreover, estrogen and calcium contribute to cholesterol metabolism. In postmenopausal women, decreased estradiol levels result in increased serum cholesterol concentration, metabolic syndrome, carotid intima-media thickness, and CVD [8].

The optimal dietary calcium intake for a beneficial effect on cardiovascular outcomes remains controversial. Several studies have reported the harmful effects of high calcium intake on CVD in men, premenopausal women, and menopausal women [5,6,9]. We found that a daily calcium intake >800 mg was associated with reduced CVD in women who had been menopausal for >10 years. However, we could not determine the recommended upper limit of calcium intake. In our study, only 2.7% of the women had a daily calcium intake >1200 mg, with no significant association with CVD events. Since there were relatively few participants with a calcium intake >1200 mg/day, we did not analyze this subgroup. The recommended dietary calcium intake varies across health associations and countries. Similar to our study, a Swedish prospective longitudinal cohort study recommended that women over fifty years old take 800 mg of calcium daily [9]. Moreover, adverse outcomes did not increase with a total calcium intake of 600–1400 mg/d. Daily calcium intake >1400 mg was associated with the risk of CVD and ischemic heart disease but not stroke. Based on the AACE and IOM, the recommended dietary allowance is 1000 mg/day for premenopausal women and 1200 mg/day for women over fifty years old [21,22]. The National Osteoporosis Guidelines Group in the UK recommends a reference calcium intake of 700 mg/day [31]. The European Menopause and Andropause Society recommends a daily intake of elemental calcium of 700–1200 mg [2]. Based on these recommendations and our findings, we suggest that the modest calcium intake for Korean women be 800–1200 mg/day and not exceed 1400 mg/day.

This study has several strengths, including its large sample size and numerous cardiovascular events. Additionally, this study was based on data from the KNHANES, which suggests that the study participants represent the general population of Korean citizens. Finally, we adjusted for several important confounding factors related to CVD risk, including age, menopausal age, insulin use, BMI, hypertension, diabetes mellitus, dyslipidemia, high alcohol intake, smoking, exercise, oral contraceptive use, and hormonal therapy use.

However, this study has several limitations. First, all medical histories and calcium intake were self-reported, which may have led to recall bias. However, other studies have also used the food frequency questionnaire to assess daily calcium intake [6,13,24], with a recent review demonstrating that it is a valid method for assessing dietary mineral intake, especially calcium intake [9]. Second, a self-reported history of angina can be further divided into unstable, stable, and variant angina. The pathophysiology of these angina types differs; accordingly, it is difficult to assume that all patients with angina have coronary artery disease. Therefore, further studies with accurate diagnoses rather than self-reported histories are warranted. Estrogen level was not reported in this study. However, we categorized women in two groups: more than 10 years post-menopause and within 10 years post-menopause. The influence of estrogen on calcium metabolism and CVD would not be different within groups. Finally, calcium intake was calculated based on the dietary consumption of calcium-containing foods, without considering supplemental calcium intake. Calcium supplements might increase the risk of CVD [20], with supplementary calcium intake >1000 mg/day being reported to be associated with CVD [5]. A report based on the 2015 KNHANES showed that although the total calcium intake from food among female participants was 467.5 mg, the total calcium intake from food and supplements was 540.1 mg [32]. Accordingly, dietary calcium supplementation may not account for major changes in the total daily calcium intake and thus may have had minimal effects in this study.

## 5. Conclusions

In conclusion, our findings suggest that appropriate dietary calcium intake (>800 mg/day) decreases the prevalence of cardiovascular events in women who have been menopausal for more than 10 years.

## Figures and Tables

**Table 1:** Clinical characteristics of the 12,348 participants stratified according to daily calcium intake.

Characteristics	Daily Calcium Intake (mg/Day)		
	Ca < 400 (*n* = 6052)	400 ≤ Ca < 800 (*n* = 4870)	Ca > 800 (*n* = 1426)
Age (years)	59.58 (0.10)	58.76 (0.10)	58.71 (0.18)
Menopause age	50.73 (0.05)	50.79 (0.05)	50.78 (0.09)
Income (%)			
Low	26.57 (0.68)	19.85 (0.69)	17.37 (1.20)
Mid-low	25.74 (0.68)	23.41 (0.75)	23.93 (1.35)
Mid-high	25.10 (0.70)	27.17 (0.75)	25.74 (1.37)
High	22.59 (0.69)	29.57 (0.85)	32.96 (1.55)
Urban area (%)	67.30 (0.85)	69.37 (0.86)	67.63 (1.57)
Education (%)			
Elementary	33.64 (0.78)	24.85 (0.74)	21.84 (1.27)
Middle school	52.41 (0.78)	54.22 (0.88)	52.67 (1.60)
High school and above	13.95 (0.61)	20.92 (0.78)	25.49 (1.47)
BMI	24.36 (0.05)	23.99 (0.05)	23.85 (0.10)
Hypertension			
Normal	32.85 (0.77)	37.73 (0.87)	40.59 (1.56)
Prehypertension	23.91 (0.65)	23.97 (0.73)	25.47 (1.41)
Hypertension	43.24 (0.79)	38.30 (0.86)	33.95 (1.45)
Dyslipidemia	27.11 (0.71)	27.17 (0.74)	23.37 (1.33)
Diabetes mellitus			
Normal	61.09 (0.8)	63.34 (0.88)	64.07 (1.6)
FPG disorder	24.01 (0.7)	24.62 (0.78)	24.05 (1.45)
Diabetes	14.9 (0.57)	12.04 (0.58)	11.88 (1)
Insulin usage	0.82 (0.14)	0.68 (0.14)	0.43 (0.16)
OCP usage	20.77 (0.61)	20.42 (0.73)	20.2 (1.29)
HT usage	15.87 (0.93)	20.37 (1.15)	19.36 (2.01)
High alcohol intake (%)	2.98 (0.28)	1.89 (0.23)	1.65 (0.40)
Smoking (%)	7.85 (0.45)	6.55 (0.47)	7.75 (0.91)
Exercise (%)			
No exercise	44.35 (0.79)	34.68 (0.82)	32.69 (1.43)
1 time per week	4.99 (0.34)	4.88 (0.38)	3.96 (0.62)
2 times per week	7.79 (0.41)	9.69 (0.51)	7.86 (0.81)
3 times per week	9.47 (0.46)	10.97 (0.54)	12.07 (1.08)
4 times per week	5.66 (0.35)	7.19 (0.46)	8.68 (0.94)
5 times per week	27.74 (0.74)	32.59 (0.82)	34.75 (1.50)

Data are presented as the mean (standard deviation) or weighted percent (standard error). OCP, oral contraceptives; HT, hormonal therapy; BMI, body mass index.

**Table 2:** Odds ratio (95% CI) for cardiovascular disease according to calcium intake in 12,348 participants.

CVD	Ca Intake (mg/Day)	Total *n*	Event *n*	Unadjusted OR	*p*-Value	Model 1 Adjusted OR	*p*-Value	Model 2 Adjusted OR	*p*-Value
All	Ca ≤ 400	6052	376	1		1		1	
	400 < Ca ≤ 800	4870	224	0.75 (0.61–0.92)	0.0071	0.81 (0.66–1.00)	0.3635	0.74 (0.53–1.03)	0.0765
	Ca > 800	1426	50	0.76 (0.52–1.11)	0.1582	0.83 (0.57–1.21)	0.6591	0.86 (0.47–1.58)	0.6264
Stroke	Ca ≤ 400	6052	207	1		1		1	
	400 < Ca ≤ 800	4870	135	0.80 (0.61–1.04)	0.0909	0.87 (0.67–1.13)	0.5544	0.76 (0.50–1.17)	0.2160
	Ca > 800	1426	25	0.57 (0.35–0.92)	0.0218	0.62 (0.38–1.01)	0.0936	0.56 (0.26–1.23)	0.1494
Angina	Ca ≤ 400	6052	58	1		1		1	
	400 < Ca ≤ 800	4870	30	0.57 (0.33–0.99)	0.0444	0.62 (0.36–1.07)	0.8413	0.55 (0.25–1.18)	0.1262
	Ca > 800	1426	4	0.31 (0.1–0.91)	0.0333	0.34 (0.11–1.00)	0.1221	0.44 (0.12–1.53)	0.1943
MI	Ca ≤ 400	6052	163	1		1		1	
	400 < Ca ≤ 800	4870	111	0.87 (0.65–1.17)	0.3655	0.95 (0.70–1.28)	0.6251	0.84 (0.52–1.36)	0.4830
	Ca > 800	1426	23	0.69 (0.42–1.15)	0.1551	0.76 (0.46–1.26)	0.3157	0.65 (0.28–1.53)	0.3276

OR, odds ratio; CVD, cardiovascular disease; MI, myocardial infarction; Model 1: adjusted for age; Model 2: adjusted for age, menopause age, income, urban area, education, insulin usage, body mass index, hypertension, diabetes mellitus, dyslipidemia, high alcohol intake, smoking, exercise, oral contraceptive usage, and hormonal therapy usage.

**Table 3:** Odds ratio (95% CI) for cardiovascular disease according to calcium intake in the group comprising <10-year postmenopausal women (*n* = 7375).

CVD	Ca Intake (mg/Day)	Total *n*	Event *n*	Unadjusted OR	*p*-Value	Model 1 Adjusted OR	*p*-Value	Model 2 Adjusted OR	*p*-Value
All	Ca ≤ 400	3419	126	reference		reference		reference	
	400 < Ca ≤ 800	3044	89	0.81 (0.57–1.14)	0.2258	0.82 (0.58–1.16)	0.0917	0.81 (0.49–1.36)	0.4313
	Ca > 800	912	30	1.23 (0.73–2.05)	0.4339	1.26 (0.75–2.10)	0.1879	1.81 (0.87–3.76)	0.1112
Stroke	Ca ≤ 400	3419	68	reference		reference		reference	
	400 < Ca ≤ 800	3044	56	0.96 (0.62–1.47)	0.8421	0.98 (0.63–1.51)	0.8952	0.87 (0.47–1.64)	0.6781
	Ca > 800	912	14	0.87 (0.45–1.70)	0.6885	0.9 (0.46–1.75)	0.7671	1.18 (0.50–2.80)	0.7057
Angina	Ca ≤ 400	3419	22	reference		reference		reference	
	400 < Ca ≤ 800	3044	12	0.72 (0.31–1.67)	0.4456	0.74 (0.32–1.72)	0.5799	0.43 (0.14–1.26)	0.1227
	Ca > 800	912	2	0.30 (0.07–1.32)	0.1109	0.31 (0.07–1.38)	0.1734	0.64 (0.13–3.24)	0.5897
MI	Ca ≤ 400	3419	54	reference		reference		reference	
	400 < Ca ≤ 800	3044	47	1.02 (0.62–1.68)	0.9243	1.04 (0.63–1.72)	0.9899	0.98 (0.48–2.00)	0.9658
	Ca > 800	912	13	1.07 (0.53–2.16)	0.8528	1.10 (0.54–2.22)	0.8309	1.56 (0.62–3.87)	0.3426

OR, odds ratio; CVD, cardiovascular disease; MI, myocardial infarction. Model 1: adjusted for age; Model 2: adjusted for age, menopause age, income, urban area, education, insulin usage, body mass index, hypertension, diabetes mellitus, dyslipidemia, high alcohol intake, smoking, exercise, oral contraceptives usage, and hormonal therapy usage.

**Table 4:** Odds ratio (95% CI) for cardiovascular disease according to calcium intake in the group comprising >10-year postmenopausal women (*n* = 4973).

CVD	Ca Intake (mg/Day)	Total *n*	Event *n*	Unadjusted OR	*p*-Value	Model 1 Adjusted OR	*p*-Value	Model 2 Adjusted OR	*p*-Value
All	Ca ≤ 400	2633	250	1		1		1	
	400 < Ca ≤ 800	1826	135	0.79 (0.61–1.02)	0.0699	0.80 (0.62–1.04)	0.3393	0.67 (0.43–1.04)	0.0714
	Ca > 800	514	20	0.46 (0.26–0.80)	0.0059	0.47 (0.27–0.82)	0.0181	0.27 (0.11–0.64)	0.0029
Stroke	Ca ≤ 400	2633	139	1		1		1	
	400 < Ca ≤ 800	1826	79	0.76 (0.55–1.06)	0.1025	0.78 (0.56–1.10)	0.3410	0.59 (0.33–1.07)	0.0817
	Ca > 800	514	11	0.39 (0.20–0.78)	0.0078	0.40 (0.20–0.81)	0.0242	0.06 (0.01–0.42)	0.0050
Angina	Ca ≤ 400	2633	36	1		1		1	
	400 < Ca ≤ 800	1826	18	0.49 (0.26–0.90)	0.0225	0.50 (0.27–0.93)	0.6982	0.80 (0.27–2.39)	0.6911
	Ca > 800	514	2	0.35 (0.07–1.66)	0.1865	0.36 (0.08–1.71)	0.3937	0.28 (0.03–2.40)	0.2444
MI	Ca ≤ 400	2633	109	1		1		1	
	400 < Ca ≤ 800	1826	64	0.85 (0.58–1.23)	0.3883	0.88 (0.60–1.28)	0.3364	0.61 (0.31–1.21)	0.1564
	Ca > 800	514	10	0.47 (0.23–0.98)	0.0426	0.49 (0.23–1.01)	0.0734	0.27 (0.11–0.64)	0.0029

OR, odds ratio; CVD, cardiovascular disease; MI, myocardial infarction. Model 1: adjusted for age; Model 2: adjusted for age, menopause age, income, urban area, education, insulin usage, body mass index, hypertension, diabetes mellitus, dyslipidemia, high alcohol intake, smoking, exercise, oral contraceptives usage, and hormonal therapy usage.

## Data Availability

We analyzed survey data from the Korean National Health and Nutrition Examination Survey (KNHANES), collected from 2005 to 2021, conducted by the Korea Disease Control and Prevention Agency. Data are publicly available through the KNHANES website (http://knhanes.cdc.go.kr).
